# Inhibition of Glycolysis Suppresses Cell Proliferation and Tumor Progression In Vivo: Perspectives for Chronotherapy

**DOI:** 10.3390/ijms22094390

**Published:** 2021-04-22

**Authors:** Jana Horváthová, Roman Moravčík, Miroslava Matúšková, Vladimír Šišovský, Andrej Boháč, Michal Zeman

**Affiliations:** 1 Department of Animal Physiology and Ethology, Faculty of Natural Sciences, Comenius University in Bratislava, Ilkovicova 6, 842 15 Bratislava, Slovakia; roman.moravcik@uniba.sk (R.M.); michal.zeman@uniba.sk (M.Z.); 2 Cancer Research Institute, Biomedical Research Center of Slovak Academy of Sciences, Dubravska cesta 9, 845 05 Bratislava, Slovakia; miroslava.matuskova@savba.sk; 3 Institute of Pathological Anatomy, Faculty of Medicine, Comenius University in Bratislava, University Hospital Bratislava, Sasinkova 4, 811 08 Bratislava, Slovakia; vladimir.sisovsky@fmed.uniba.sk; 4 Department of Organic Chemistry, Faculty of Natural Sciences, Comenius University in Bratislava, 842 15 Bratislava, Slovakia; andrej.bohac@uniba.sk

**Keywords:** glycolysis, PFKFB3, PFK15, cancer, chronotherapy, HUVEC, DLD1 cells

## Abstract

A high rate of glycolysis is considered a hallmark of tumor progression and is caused by overexpression of the enzyme 6-phosphofructo-2-kinase/fructose-2,6-biphosphatase 3 (PFKFB3). Therefore, we analyzed the possibility of inhibiting tumor and endothelial cell metabolism through the inhibition of PFKFB3 by a small molecule, (*E*)-1-(pyridin-4-yl)-3-(quinolin-2-yl)prop-2-en-1-one (PFK15), as a promising therapy. The effects of PFK15 on cell proliferation and apoptosis were analyzed on human umbilical vein endothelial cells (HUVEC) and the human colorectal adenocarcinoma cell line DLD1 through cytotoxicity and proliferation assays, flow cytometry, and western blotting. The results showed that PFK15 inhibited the proliferation of both cell types and induced apoptosis with decreasing the Bcl-2/Bax ratio. On the basis of the results obtained from in vitro experiments, we performed a study on immunodeficient mice implanted with DLD1 cells. We found a reduced tumor mass after morning PFK15 treatment but not after evening treatment, suggesting circadian control of underlying processes. The reduction in tumor size was related to decreased expression of Ki-67, a marker of cell proliferation. We conclude that inhibition of glycolysis can represent a promising therapeutic strategy for cancer treatment and its efficiency is circadian dependent.

## 1. Introduction

Despite great advances in prevention and treatment, cancer remains one of the most common causes of death worldwide. In the last years, several different anticancer treatments have been developed. However, their success is still limited, therefore, new strategies are needed. Cell metabolism, especially aerobic glycolysis, represents a promising area of anticancer treatment [1,2]. Several studies have suggested that the inhibition of specific aspects of cell metabolism can reduce cancer progression [3] or improve the results of chemotherapy, with limited negative effects on host metabolism and health [4]. 

Cancer cells are characterized by a high rate of glycolytic flux, even in the presence of sufficient oxygen supply, a phenomenon known as the Warburg effect [5]. This metabolic alteration allows cancer cells to proliferate at an abnormally high rate, even under hypoxic conditions. The increased level of glycolysis provides anabolic substrates to support cell proliferation and migration, as well as the high ATP demand of tumor cells. A rate-limiting step in glucose metabolism is the conversion of fructose-6-phosphate to fructose-1,6-bisphosphate (F1.6P2) by the enzyme phosphofructokinase 1 (PFK1) [6]. The enzyme 6-phosphofructo-2-kinase/fructose-2,6-bisphosphatase 3 (PFKFB3) catalyzes the synthesis of fructose-2,6-bisphosphate (F2.6P2), which is an allosteric activator of PFK1 and the most effective stimulator of glycolysis [7]. Protein levels of the glycolysis regulator PFKFB3 are overexpressed in a variety of cancers, and expression of this enzyme is strongly correlated with a poor prognosis [4]. Moreover, PFKFB3 is associated with the process of angiogenesis through the stimulation of vessel sprouting and branching mediated by endothelial cells [1]. In this way, inhibition of glycolysis provides an attractive target in the field of angiogenesis research. 

The activity of PFKFB3 can be blocked by various novel compounds, including chalcones, phenoxyindoles, aminoquinoxalines, and peptides [8]. The first described inhibitor of PFKFB3, (*E*)-3-(pyridin-3-yl)-1-(pyridin-4-yl)prop-2-en-1-one (3PO), exhibits potent antitumor activity [6], reduces the intracellular concentration of F2.6P2, and suppresses the level of glycolysis in cells. Problems with the low solubility of 3PO led to the development of several analogues, among which (*E*)-1-(pyridin-4-yl)-3-(quinolin-2-yl)prop-2-en-1-one (PFK15) [9] and its trifluoromethyl derivative (*E*)-1-(4-pyridinyl)-3-(7-(trifluoromethyl)-2E-quinolinyl)-2-propen-1-one (PFK158) seem to be the most promising [10]. PFK15 results from the substitution of a quinolinyl group for a pyridine ring in the structure of 3PO and displays higher selectivity than 3PO. PFK15 causes a reduction in glucose uptake and lactate production in different types of cells [9]. Another glycolytic inhibitor, PFK158, shows even higher efficiency in vitro and in vivo, and was included in the first phase of clinical trials [11]. 

In addition to tumor cells, an enhanced preference for aerobic glycolysis is also observed in endothelial cells and cells present in the tumor microenvironment [12]. Endothelial cells play an important role in angiogenesis, especially in vessel sprouting, which is a highly coordinated process regulated by chemical signals produced by tumor cells in the rapid growth phase. Upon the induction of sprouting by growth factors, quiescent endothelial cells become active, increase their level of glycolysis, and start to proliferate and migrate. This process is known as an angiogenic switch [13]. These newly formed vessels deliver nutrients and oxygen to tumor tissues, thereby contributing to cancer development. 

Cellular metabolism, both within the tumor microenvironment and in the host tissue, is under circadian control [14]. Circadian clocks maintain cell-autonomous circa 24 h oscillations through the circadian transcriptional/translational feedback loop consisting of transcription factors CLOCK and BMAL 1, which control the transcription of canonical clock genes and hundreds of clock-controlled genes. As a result, metabolism in healthy cells exhibits distinct circadian oscillations, which can be disrupted in cancer cells [15,16,17]. Clinical data show that circadian expression of core clock genes is deregulated in several human cancers [18], including colorectal cancer [19,20]. Circadian clocks control a wide array of metabolic processes, including glycolysis, which exhibits a distinct circadian pattern. Additionally, PFKFB3 shows circadian expression at mRNA and protein level, which is controlled predominantly by transcription factor CLOCK, as shown in tongue cancer cell line [21]. Moreover, the study showed that inhibition of PFKFB3 at its peak levels by glycolysis inhibitor 3PO resulted in a significant decrease of cell proliferation and lactate production. Therefore, specific targeting of metabolic processes in a time-dependent manner (chronotherapy) can offer a potential advantage in cancer treatment [22]. 

Approaches aimed at simultaneously inhibiting cancer cell proliferation and angiogenesis represent an attractive possibility to limit tumor progression. Therefore, in the present study, we focused on the mechanisms underlying the effects of PFK15 on a cancer cell line and on primary endothelial cells. We analyzed signaling pathways that regulate the key processes of cell proliferation, migration, and apoptosis to examine whether both cell types respond to PFKFB3 inhibition in a similar way. In the next step, we explored the consequences of glycolysis inhibition on tumor progression induced by subcutaneous administration of human colorectal carcinoma-derived cells to athymic mice. Treatment with PFK15 was performed at two different times of day to explore whether chronotherapy represents a promising approach in relation to glycolysis inhibition. 

## 2. Results

### 2.1. PFK15 Reduced Cell Proliferation 

**Figure 1:**
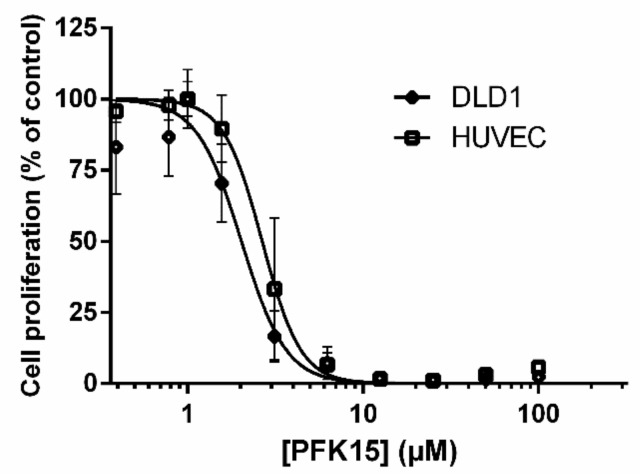
Dose-dependent effect of PFK15 treatment for 3 days on the proliferation of DLD1 and HUVEC cells. Cells were incubated with PFK15 in a concentration range from 0.39 to 100 µM for 3 days. Data are expressed as the percentage of proliferation of PFK15-treated cells compared to untreated cells. Each point represents the mean of quadruplicate experiments. IC_50_ values were calculated from a dose–response curve as the concentration of PFK15 yielding 50% of control cell survival.

In the first part of our study, we investigated the ability of PFK15 to inhibit the proliferation of DLD1 and HUVEC cells in vitro. Our results showed that PFK15 inhibited the growth of HUVEC (IC_50_ 2.6 µM) and DLD1 cells (IC_50_ 2.0 µM) with a similar efficiency (Figure 1). Because PFK15 inhibited DLD1 and HUVEC proliferation at a similar level, we can expect that the same molecular processes were affected by this treatment. 

**Figure 2:**
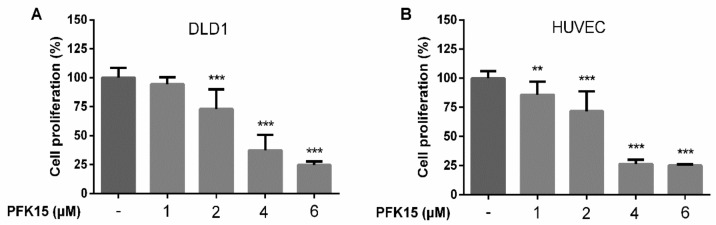
Proliferation of DLD1 (**A**) and HUVEC (**B**) cells was assessed after PFK15 treatment. Cell proliferation was determined based on bromodeoxyuridine incorporation into DNA. DLD1 and HUVEC cells were treated with PFK15 in a concentration range 1–6 µM for 24 h. Alterations in cell proliferation were compared to the untreated control group. Data are presented as the mean ± SEM of three independent experiments. ** *p* < 0.01, *** *p* < 0.001 significant difference compared to untreated control cells.

Subsequently, we analyzed changes in DLD1 (Figure 2A) and HUVEC (Figure 2B) proliferation after exposure to PFK15 for 24 h based on the incorporation of BrdU during DNA synthesis. Exposure to PFK15 in a concentration range from 1 to 6 µM induced a dose-dependent reduction in cell proliferation compared with control cells. 

**Figure 3:**
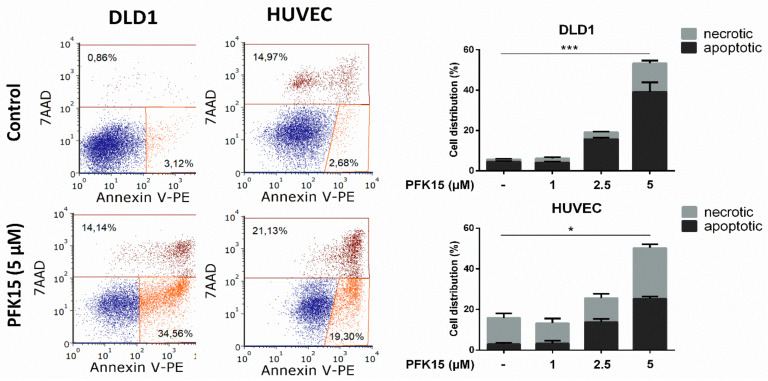
PFK15 induces apoptosis in DLD1 and HUVEC cells cultured in the presence of PFK15 for 48 h. Annexin V assay and subsequent flow cytometric analysis were used for cell death evaluation. Left panel: representative dot-plots from flow cytometric analysis. Right panel: results are presented as mean of triplicates ± SEM. One out of three independent experiments is presented. * *p* < 0.05, *** *p* < 0.001.

Annexin V assay revealed the capacity of PFK15 to induce apoptosis. After incubation for 48 h in the presence of the drug, we observed a significant, concentration-dependent increase of apoptotic cells both in DLD1 and in HUVEC cultures (more than 8-fold at 5 µM concentration of PFK15) (Figure 3). 

**Figure 4:**
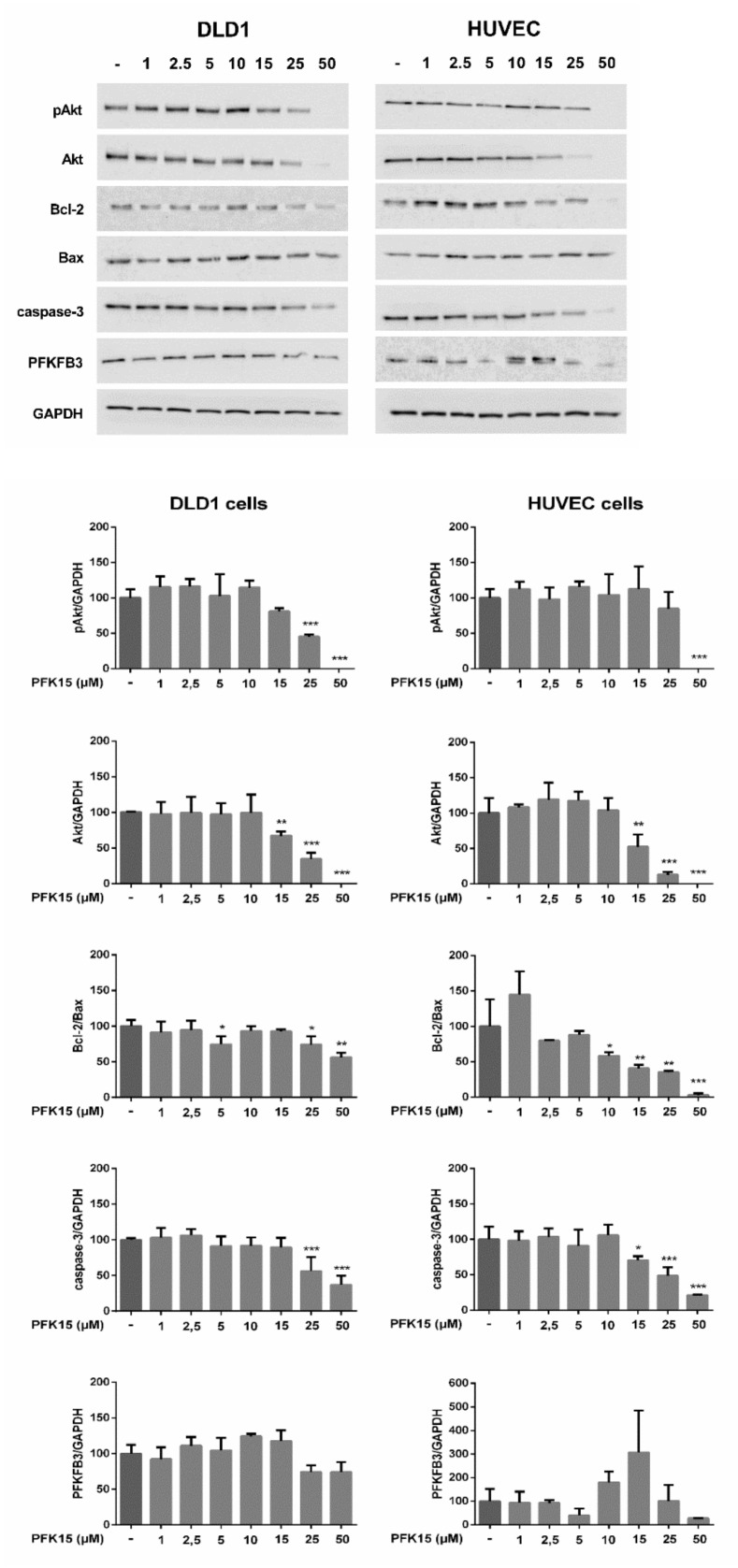
Effect of PFK15 on the level of proteins associated with cell proliferation, apoptosis, and glucose metabolism in DLD1 and HUVEC cells. Cells were treated with PFK15 in a concentration range from 1 to 50 µM for 24 h. Western blots were quantified by densitometry and expressed as the percentage of protein expression compared to the reference protein GAPDH used as a loading control. Untreated cells incubated only in medium served as the control (100%). Data represent the mean ± SEM of three independent experiments. * *p* < 0.05, ** *p* < 0.01, *** *p* < 0.001 significant difference compared to untreated control cells.

To analyze the molecular mechanism of PFK15-induced inhibition of cancer progression, we determined changes in the expression of key proteins in the underlying signaling pathways. Among the pathways that regulate cellular processes, PI3K/Akt plays an important role in the control of essential cellular functions, such as proliferation, migration, and apoptosis. We examined the effect of PFK15 on the PI3K/Akt signaling by determination of the total and phosphorylated forms of the Akt protein. As shown in Figure 4, Akt phosphorylation was attenuated after PFK15 treatment in both cell types, with a stronger effect observed in DLD1 compared with HUVEC cells. Both DLD1 and HUVEC cells treated with the higher dose of PFK15 showed a decrease in Akt content. We subsequently analyzed changes in the levels of the antiapoptotic protein Bcl-2 and proapoptotic protein Bax. We found a significant reduction in Bcl-2 expression in both cell types (in HUVEC at PFK15 >15 µM and in DLD1 at PFK15 >25 µM), while the expression of Bax was unchanged after PFK15 treatment compared to control. As a result, the Bcl-2/Bax ratio was downregulated in comparison with control cells, with a stronger effect observed in HUVEC. Moreover, PFK15 induced downregulation of caspase-3 in both cell types (Figure 4). The protein level of the key glycolytic enzyme PFKFB3 was not significantly affected by PFK15 treatment. In DLD1 cells, the high concentration of PFK15 tended to decrease the expression of this enzyme, while in HUVEC, the pattern was highly variable and did not reach statistical significance.

**Figure 5:**
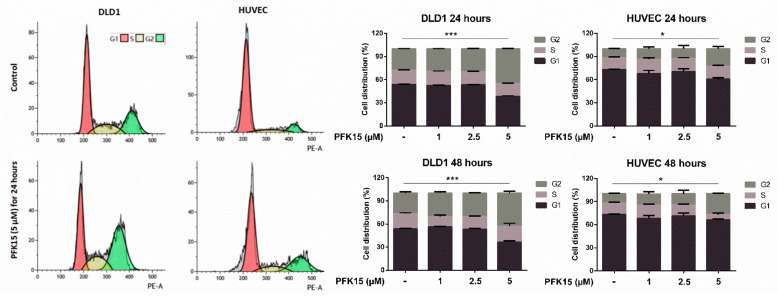
PFK15 induced arrest in the G2 phase of the cell cycle. Cells were cultured in the presence of PFK15 for 24 and 48 h. DNA was stained by propidium iodide and analyzed by flow cytometry. Left panel: representative pictures of cell cycle analysis. Right panel: results are presented as mean of triplicates ± SEM. One out of three independent experiments is presented. * *p* < 0.05, *** *p* < 0.001.

Flow cytometric analysis showed changes in the cell cycle as a result of PFK15 treatment. We observed a significant accumulation of cells in G2 phase and decrease in G1 and S after incubation with 5 µM PFK15 in both analyzed cell types (Figure 5). 

**Figure 6:**
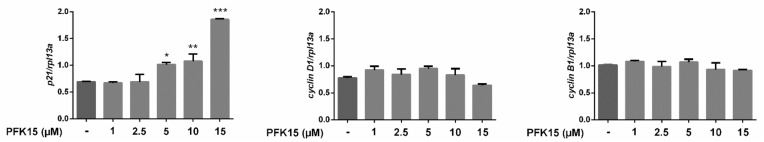
Effect of PFK15 on the expression of genes regulating the cell cycle in DLD1 cells. DLD1 cells were treated with PFK15 in a concentration range of 1–15 µM for 24 h. Expression of mRNA was analyzed by real-time PCR and normalized to the level of rpl13a. Results are displayed as mean ± SEM (*n* = 3). * *p* < 0.05, ** *p* < 0.01, *** *p* < 0.001.

We then analyzed the expression of genes involved in cell cycle control in DLD1 cells. Expression of *p21* was upregulated by PFK15 treatment at 10 and 15 µM. Expression of cyclin D1 slightly decreased after PFK15 treatment, but expression of cyclin B1 remained unchanged compared to the control (Figure 6). 

### 2.2. PFK15 Inhibits Tumor Growth In Vivo 

Because PFK15 attenuated proliferation and reduced Bcl-2/Bax ratio in both cancer and activated endothelial cells, we investigated the in vivo effects of PFK15 on tumor growth. We treated nude mice bearing tumors induced by DLD1 cells with PFK15 at 25 mg/kg or a vehicle a total of five times over 12 days. 

**Figure 7:**
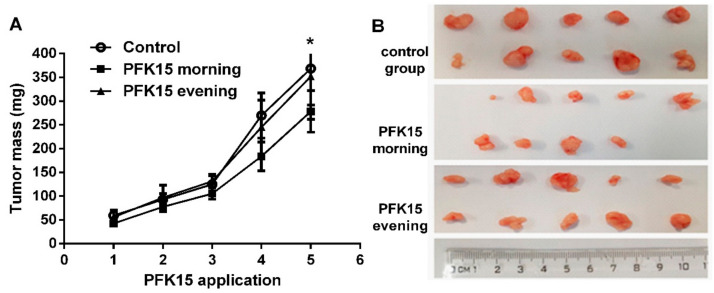
Changes in tumor size after PFK15 administration (control, morning, and evening groups). (**A**) Changes in the size of tumors were assessed after each PFK15 administration. The graph presents the changes in tumor mass in the control group compared to the groups that received morning or evening PFK15 treatment over 12 days (in total, 5 administrations). On day 14, tumor-bearing mice were sacrificed, and excised tumors were measured. Results are expressed as mean ± SEM for the control (*n* = 5) and morning- and evening-treated groups (*n* = 6) (*p* < 0.05). (**B**) Photographs of tumors extirped from animals treated with the vehicle (control group) or PFK15 in the morning or evening. A ruler was used to measure (in mm) extirpated tumors after morning PFK15 treatment, evening PFK15 treatment, or vehicle administration.

The results of the treatment on tumor growth are illustrated in Figure 7. Measurement of tumor size during the 14-day period revealed an anticancer effect of PFK15 in the group treated in the morning, in which we observed attenuated tumor growth. In animals treated in the evening, we did not observe any significant differences in tumor size compared to vehicle-treated animals. The body weight of animals was not affected by treatment with PFK15. The tumors extirpated from each treated group are shown in Figure 7B. 

**Figure 8:**
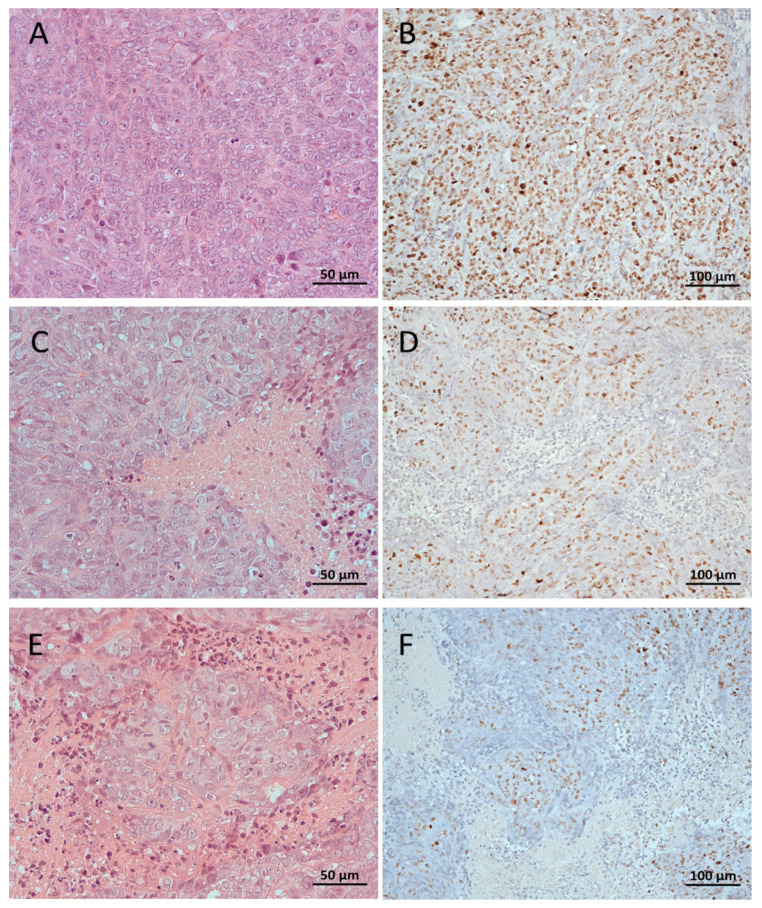
Subcutaneous xenografts induced by human colorectal carcinoma cells formed in athymic mice without PFK15 administration (**A**,**B**), with PFK15 applied in the morning (**C**,**D**), and with PFK15 applied in the evening (**E**,**F**). Hematoxylin and eosin, 400× (left), and immunohistochemical staining with monoclonal antibody Ki-67, Polymer-Px (EnVision), and hematoxylin, 200× (right). **A**,**B**: with strongly intense immunostaining in >80% of the cells; these cases contained regressive changes (necrosis) of the tumor parenchyma in the range of 11–25% of the area. **C**,**D**: the staining intensity: 2, moderately, to 3, strongly; the quantity of cells stained: 2, 11–50%; an immunoreactive total score: 4–6, moderate; regressive changes (necrosis) of the tumor parenchyma in the range of 26–49% of the area. **E**,**F**: an immunoreactive total score: 6, moderate, to 9, strong; regressive changes (necrosis) of the tumor parenchyma in the range of ≥50% of the area.

Tumor tissues were evaluated by histological examination with hematoxylin and eosin and immunohistochemical staining. The results revealed that the expression of proliferation marker Ki-67 was higher in untreated tumor tissues (control group) compared with the morning or evening PFK15-treated groups. In the morning group, three of six cases showed strong Ki-67 immunoreactivity while the remaining three cases showed moderate immunoreactivity (Figure 8). Evening PFK15 administration showed strong immunoreactivity in five of six tumor tissue samples and moderate immunoreactivity in one case (Table 1). 

Regressive changes of tumor parenchyma were more pronounced in PFK15-treated groups as compared with controls and were found in the range 11–25% of the area in all cases. In the treated mice, the regressive changes were identified in the lowest range (1–10% of the area) in 33% in the morning group and in 17% cases in the evening group. In the next range of regression (26–49%), 50% cases were identified in the morning group and 17% cases in the evening group. The most prominent regressive changes (≥50% of the area) were found in 17% of the morning group and in 66% of the evening group. Taken together, our data indicate that PFK15 treatment induced regressive changes in tumor parenchyma and reduced expression of proliferation marker Ki-67 in the morning and evening treated groups as compared with vehicle-treated animals (Figure 8).

Expression of the marker of endothelial cells PECAM-1 (platelet endothelial cell adhesion molecule-1) did not differ among experimental groups. 

In summary, in the present study we investigated the possible anticancer effects of glycolysis inhibition through PFK15 and the underlying mechanisms of its action on human endothelial cells HUVEC and on a human colorectal cancer cell line DLD1. We found that PFK15 treatment suppressed proliferation and promoted apoptosis of both cell types in vitro. Our subsequent in vivo study on athymic mice revealed an inhibitory effect of PFK15 on the progression of tumors induced by DLD1 cells after morning but not evening treatment. 

## 3. Discussion

In the present study, we investigated the anticancer effects of glycolysis inhibition through PFK15 and the underlying mechanisms of its action on a human colorectal cancer cell line and human endothelial cells (DLD1 and HUVEC, respectively). Our study for the first time explored the effects of glycolysis inhibitor PFK15 on colorectal adenocarcinoma cell line and compared results with those obtained in primary endothelial cells. We found that PFK15 treatment suppressed proliferation and promoted apoptosis of both cell types in vitro. Our subsequent in vivo study on athymic mice revealed an inhibitory effect of PFK15 on the progression of tumors induced by DLD1 cells after morning but not evening treatment. 

Administration of PFK15 reduced the proliferation of DLD1 and HUVEC cells in a dose-dependent manner. The IC_50_ values calculated for both cell types were similar and close to 2 µM. HUVEC and DLD1 cells are of human origin and are characterized by an increased level of glucose metabolism; therefore, the effects of PFK15 could be mediated by similar mechanisms in both cell types. The inhibitory effect of PFK15 is important for cancer treatment because the drug can simultaneously suppress the proliferation of cancer cells and endothelial cells, which are responsible for the formation of new vessels and provide nutrients and oxygen to the proliferating tumor cells [1]. Previously published data suggest that the effects of PFK15 depend on the cell type, and IC_50_ values ranging from 6.6 to 10.6 µM have been reported for the MKN45 and BGC gastric cancer cell lines, respectively [3]. We confirmed the effect of PFK15 using another colorectal cell line, HCT116 with the IC_50_ value 1.34 µM (data not shown). The similar results in HCT116 and DLD1 cells demonstrate that the inhibitory effect of PFK15 is not specific only for DLD1 cells. In HCT116 cells, p53 was found to regulate glycolytic metabolism, and therefore using HCT166 -/- cells may further elucidate p53 function in this process [23]. In Jurkat leukemia cells, the IC_50_ value was lower (2.42 µM) compared with cancer cell line MKN45 and BGC, respectively. The value as low as 0.72 µM was reported in H522 adenocarcinoma cells. Our results show that both cell types tested in the current study are sensitive to glycolysis inhibition and did not differ substantially, even though HUVEC represents normal healthy human cells and DLD1 cells derived from colorectal adenocarcinoma represent a cell line with unlimited cell proliferation. These findings are in line with published data demonstrating decreased glucose uptake and F2.6P2 production in other cell types [3]. Both lines of evidence suggest a suppressive effect of PFK15 treatment on ATP-consuming processes, such as proliferation, migration, and cell differentiation. Moreover, it is possible that the effects of glycolysis inhibitors can be enhanced by other anticancer drugs or administration in combination with inhibitors, which suppress angiogenesis, cell metabolism, and autophagy [24]. Indeed, in our recent study, we found that simultaneous treatment of HUVEC with the glycolysis inhibitor 3PO and multikinase inhibitor sunitinib attenuated cell proliferation and migration and potentiated the antiangiogenic action [25].

To understand the mechanisms underlying the effects of PFK15 on HUVEC and DLD1 cells, we investigated signaling pathways controlling cell proliferation, migration, and apoptosis. PFK15 treatment decreased levels of the protein kinase Akt and reduced its phosphorylation in both cell types. Similar effects have been reported in breast cancer cells treated with the allosteric Akt inhibitor MK-2206 in combination with salinomycin, a potential inhibitor of tumor stem cell growth [26]. The authors concluded that cancer cells treated with salinomycin were more sensitive to MK-2206 because they displayed a total reduction in Akt. An inhibitory effect on Akt phosphorylation was also observed after PFKFB3 silencing in a breast cancer cell line, confirming the potential of PFKFB3 inhibitors to act as metabolic regulators [27]. 

Signaling pathway PI3K/Akt represents the cascade of protein kinases, which regulate cell growth and proliferation and stimulate glycolytic activity [28]. Akt signaling induces transcription of the glucose transporter GLUT1 and phosphorylation and activation of PFKFB3, thereby increasing the kinase activity of this enzyme. Reduced Akt phosphorylation and subsequent glycolysis activity have been shown to suppress the cell growth and proliferation rate of Chinese hamster ovary cells [29]. Deregulation of the signaling pathway PI3K/Akt/mTOR has been demonstrated in several types of cancer, such as breast cancer and colorectal cancer [30]. Because glycolysis is closely related to AMP-activated protein kinase (AMPK), which allows cells to proliferate only under conditions of sufficient energy supply, changes in AMPK activation can be of great importance. AMPK kinase regulates cellular energy homeostasis through the activation of glucose and fatty acid oxidation [31]. A more pronounced effect on AMPK phosphorylation was observed after 12 h in comparison to two hours of PFK15 treatment in rhabdomyosarcoma cells. These results also demonstrate the time-dependent effects of PFK15 treatment [32]. 

Tumor progression reflects a balance between cell proliferation and apoptosis. Therefore, in addition to cell proliferation, we explored the effect of PFK15 on apoptosis in both cell types. Annexin V assay demonstrated a significant proapoptotic effect of PFK15 in both analyzed cell types. Expression of the antiapoptotic Bcl-2 protein decreased with an increasing concentration of PFK15, while expression of the proapoptotic protein Bax did not change significantly. The differential regulation of Bcl-2 and Bax expression resulted in a decreased Bcl-2/Bax ratio, which is a crucial factor favoring increased apoptosis [33]. The apoptotic effect of PFK15 could be explained by cytotoxic calcium overload and subsequent cell death. Glycolysis inhibition reduces the amount of ATP required for plasma membrane calcium ATPases, responsible for the low intracellular calcium observed in pancreatic cells [34], but this effect was not present in noncancerous human pancreatic stellate cells. Our previous study showed induction of apoptosis in cancer DLD1 cells due to an increased intracellular concentration of calcium released from the endoplasmic reticulum via enhanced expression of inositol 1,4,5-trisphosphate (IP3R1) receptors, but this effect was not observed in normal endothelial EA.hy 926 cells [35].

We found reduced expression of effector caspase-3 after PFK15 treatment, which can be explained by cleavage of the total form of caspase to the active phosphorylated form. The Bcl-2 protein can stabilize the mitochondrial membrane and block the release of cytochrome C, while the proapoptotic protein Bax contributes to the release of cytochrome C from the mitochondria, thereby assisting in the activation of caspase-3 and -9 [36]. Thus, our data are in line with published results, suggesting that PFKFB3 inhibits apoptosis and inhibition of PFKFB3 suppresses cell cycle progression and accumulation of cells in G0/G1phase [3]. PFKFB3 is upregulated at the transition from the G1 to S phase of the cell cycle, promoting cell division in conditions of sufficient nutrient supply [37]; therefore, suppressed PFKFB3 activity is related to decreased cell division. In contrast to abovementioned study [3], we observed arrest in G2 phase after exposition to PFK15 for 24 or 48 h. We suppose that sensitivity of cells to PFK15 and concentration of the drug play a role in the impact on cell cycle. This phenomenon was also observed, for example, in berberine and its derivatives, when low concentrations induced G0/G1 arrest while higher concentrations induced accumulation of cells in G2/M phase [38,39,40,41]. 

Moreover, the proapoptotic effect of suppressed PFKFB3 activity can be explained by increased expression of cyclin-dependent kinase inhibitor 1B (p27) in cancer cells following PFKFB3 inhibition. Studies have supported the role of p27 in the regulation of cell proliferation and apoptosis, and increased levels of this protein were observed in cancer cells after PFKFB3 knock-out [27]. 

Increased PFKFB3 is considered a prognostic marker of cancer [4] because it is related to Ki-67 expression, which is a marker of cell proliferation. In our study, we did not find significant changes in PFKFB3 expression in either of the tested cell types treated with PFK15 for 24 h, indicating that the treatment predominantly affects the activity of this enzyme and not its expression. In lung adenocarcinoma cells, levels of PFKFB3 were downregulated after PFK15 treatment, and upregulated expression of PFKFB3 in breast cancer tissues assessed by immunohistochemistry was found to be associated with poor patient prognosis [27]. The results obtained in the current study indicate that different cell types can differ in their sensitivity to PFK15.

In the next step, we focused on characterizing the expression of genes involved in cell cycle control in DLD1 cells treated with PFK15. We found a dose-dependent increase in p21 mRNA after PFK15 treatment. Because p21 negatively affects the activity of cyclin and cyclin-dependent kinases [42], we next quantified cyclin D1 and B1 mRNA. We found a trend toward decreased expression of cyclin D1 mRNA after treatment with PFK15 at the highest concentrations. This cytostatic effect of p21 could be observed after p53 binding to its promotor, which contains several binding sites for p53, and for c-Jun, respectively. Tumor suppressor p53 induces cell cycle arrest after DNA damage, and conversely, activation of c-Jun induces apoptosis [43]. Since p53 and c-Jun compete for binding site on p21 promotor, in HCT116 p53 -/- cells c-Jun may downregulate p21 expression. Induction of p21 by p53 may be also blocked by transcription factor myc, which promotes cell growth and proliferation [44]. Overexpression of p21 in prostate cancer cells has been reported to reduce tumor volume in mice and induce apoptosis, with similar results found in cervical cancer cells [45]. Moreover, blocking PFKFB3 activity with PFK15 resulted in a significant reduction in cyclin D1 expression in tumor cells isolated from lung adenocarcinoma after 24 h [4].

As the in vitro data revealed antiproliferative and apoptotic effects of PFK15 on cancer and endothelial cells, we investigated the efficiency of PFK15 in reducing growth and progression of tumor xenografts in athymic mice implanted with DLD1 cells. The dose of the drug was chosen based on previously published studies [3,9]. PFK15 was administrated either in the morning, two hours after the onset of the light phase, or in the evening, two hours before the onset of the dark phase. We found a reduced tumor mass in the group that received the morning PFK15 treatment, while no significant effects were recorded after the evening treatment. The size of the tumor was reduced after 12 days of the morning treatment. It is possible that longer treatment with PFK15 can have a more profound effect on tumor progression, but it could also cause necrosis due to an insufficient nutrient supply. Administration of PFK15 for 15 days to nude mice with an induced gastric tumor resulted in a significant decrease in tumor size after 6 days [3], suggesting specific efficacy of the treatment depending on the cell type. Although we found a reduction in tumor mass, we did not record any changes in the body weight of animals or their general appearance, suggesting that PFK15 is tolerated and can suppress tumor growth without serious side effects. Moreover, it was confirmed that the viability of normal epithelioid cells was suppressed less than 10% compared to cancer cells after PFK15 treatment [4]. Obviously, more data are needed to prove the tolerability of this treatment, but it seems that specific inhibition of PFKFB3 does not substantially interfere with physiological processes at the system level; therefore, it represents a promising target for cancer treatment. 

Marker Ki-67 is considered to be a prognostic marker for cancer due to its increased expression in tumor tissues, which is directly correlated with tumor differentiation, TNM classification, and five-year survival [4]. Our results suggest that the reduction in tumor size after morning PFK15 treatment is related to decreased expression of Ki-67, which plays a role in cell proliferation. Increased Ki-67 expression has been shown to be a negative prognostic marker of patient survival with pulmonary adenocarcinoma [4].

The different effects of morning and evening treatment with PFK15 on tumor growth and progression indicate that the chronopharmacological approach can be helpful in cancer treatment. Our data are in line with the results of recent study [21], which reported circadian control of PFKFB3 mRNA levels in human tongue carcinoma cells, with increased levels in the early light (passive) phase of a 24 h day, with peak levels between ZT5 and ZT9 (ZT = Zeitgeber Time, ZT0 indicates the beginning of the light phase), and lowest levels between ZT17 and ZT21. PFKFB3 suppression by the glycolytic inhibitor 3PO at ZT7 led to enhanced glycolysis inhibition and reduced lactate production [21]. Significant inhibition of tumor progression after morning glycolysis inhibition is in line with our results and data obtained in another animal model, in which nude mice were implanted with breast tumor cells into the femoral artery [46]. These authors observed an increased uptake of arterial glucose by tumor cells (Warburg effect) and the release of lactate into the blood. Lactate production reflected an increase in glycolysis during the passive phase, reaching a peak two hours before lights off and a minimum in the middle of the active phase. Moreover, circadian oscillations in [^3^H] thymidine incorporation and total DNA content suggest that the number of tumor cells increased during the light phase due to increased cell proliferative activity. In contrast, during the dark phase, the number of cells was lower, indicating cell apoptosis with a concomitant reduction in cell proliferation [46]. These results are consistent with previous studies suggesting that tumor growth shows circadian variations [47]. Because PFK15 predominantly affects proliferating cells, which are activated especially during the passive phase of the day [46], we hypothesize that the more pronounced effect of PFK15 observed in the group that received morning administration of PFK15 is related to circadian processes peaking during the passive phase of the day. 

## 4. Materials and Methods

### 4.1. Cell Culture and Cultivation 

Human colorectal carcinoma cell line (DLD1; CCL-221, ATCC, USA) was cultured in RPMI GlutaMAX medium (Gibco, Gaithersburg, MD, USA) supplemented with 10% FBS (Biosera, Nuaille, France), 100 U/mL penicillin, and 100 μg/mL streptomycin (Biosera). Human umbilical vein endothelial cells (HUVEC) were isolated from fresh umbilical cords, as previously described [24], and cultured in complete ECGM medium (PromoCell, Heidelberg, Germany) supplemented with 100 U/mL penicillin and 100 μg/mL streptomycin (Biosera). Cells were cultivated at 37 °C in a humidified incubator (Heal Force, Shanghai, China) containing 5% CO_2_. 

### 4.2. Drug Preparation 

The PFK15 inhibitor was prepared in four synthetic steps by Biomagi Inc. (Bratislava, Slovak Republic), and the stock solution was prepared in 100% dimethyl sulfoxide (DMSO) (Sigma-Aldrich, Saint-Louis, MO, USA) at a concentration of 10 mM. The stock solution was subsequently diluted in RPMI or ECGM medium for in vitro experiments. Controls represent medium with DMSO at the highest concentration used for PFK15 dissolving. In addition, PFK15 was suspended in 5% DMSO, 45% PEG300, 1% Tween80, and 49% H_2_O for in vivo experiments. 

### 4.3. IC_50_ of PFK15

Cells were seeded in a 96-well plate with serial dilutions of PFK15 (starting at 100 µM containing 1% DMSO) at a density of 3 × 10^3^ cells/well. Cells were incubated for 3 days, and then 10 µM of MTS reagent was added, according to the manufacturer’s instructions (CellTiter 96^®^ Aqueous MTS Reagent, Promega, Madison, WI, USA). The absorbance was measured at a wavelength of 490 nm (Apollo LB913, Berthold Technologies, Wien, Austria). The half maximal inhibitory concentration (IC_50_) was evaluated using Graph Pad Prism 6 software (Graph Pad, San Diego, CA, USA).

### 4.4. Colorimetric BrdU Assay

Cell proliferation was determined by BrdU assay based on bromodeoxyuridine incorporated during DNA synthesis. Briefly, cells were seeded in a 96-well plate at a density of 1 × 10^3^ cells/well. After 24 h, cells were treated with 1, 2, 4, or 6 µM of PFK15 containing 1% DMSO. A colorimetric BrdU assay (Roche, Meylan, France) was performed after 24 h according to the manufacturer’s manual. 

### 4.5. Detection of Apoptosis by Annexin V Assay

Cells were seeded on 24-well plates (4  ×  10^4^/well). The next day, PFK15 was added at required concentrations to the respective wells, and cells were treated for 48 h. Harvested cells (also the ones from supernatant) were washed in PBS. The pellet was subsequently resuspended in binding buffer containing PE-conjugated Annexin V (eBioscience, San Diego, CA, USA) and incubated for 15 min at room temperature, protected from light. 7-Amino-Actinomycin D (7AAD; 2 μg/mL) was added to detect dead cells. Analysis was performed on BD FACSCanto™ II flow cytometer (BD Bioscience, San Jose, CA, USA), data were analyzed with FCS Express program.

### 4.6. Analysis of Cell Cycle 

Cell cycle was determined by flow cytometric measurement of DNA content. Cells were seeded on a 12-well plate (8  ×  10^4^ for DLD1; 12  ×  10^4^ for HUVEC) and cultured overnight. Cells were subsequently cultured in the presence of PFK15 at required concentration for 24 and 48 h. For analysis, cells were collected by trypsinization, washed with cold PBS, resuspended in 300 µL PBS with 0.05% Triton X-100 and RNA-se A (100 µg/mL), and incubated for 20 min at 37 °C. Afterwards, cells were cooled on ice for 1 min and subsequently, propidium iodide (PI, 40 µg/mL) was added. Cells were analyzed using a CytoFLEX S flow cytometer (Beckman Coulter, Brea, CA, USA). Forward/side light scatter characteristic was used to exclude the cell debris from the analysis. Doublets were excluded from analysis based on 690/50 peak area vs. peak height, lin 585/42 was used for DNA cell cycle histogram. Data were analyzed by Kaluza software (Beckman Coulter).

### 4.7. Western Blot 

Cultured DLD1 and HUVEC cells were lysed in lysis buffer containing 0.02 M Tris, 0.15 M NaCl, 0.09 M KCl, 0.002 M EDTA, 5% Igepal, 0.5% Triton-X 100, 0.0002 M Na3VO4, 0.01 M NaF, and 0.1% protease inhibitor (Sigma-Aldrich, Saint-Louis, MO, USA). Total protein concentration was determined using the bicinchoninic acid assay (BCA assay kit; Sigma-Aldrich). Proteins were separated by SDS-PAGE (Bio-Rad Laboratories, Hercules, CA, USA) and transferred to a 0.22 µm or 0.45 µm nitrocellulose membrane (Thermo Fisher Scientific, Waltham, MA, USA). The membrane was blocked with 5% bovine serum albumin (BSA; BioWest, Nuaillé, France) for 1 h at room temperature to prevent nonspecific binding of antibodies. The blots were incubated with anti-p 44/42 MAPK (ERK1/2; 1:1000), anti-phospho-p44/42 MAPK (pERK1/2; 1:1000), anti-Akt, anti-phospho-Akt (1:2000), anti-caspase-3 (1:1000), anti-PFKFB3 (1:1000), anti-Bcl-2 (1:1000), anti-Bax (1:1000), and anti-GAPDH (1:2000) antibodies, followed by incubation with goat anti-rabbit HRP (1:2000) or horse anti-mouse HRP (1:2000) secondary antibodies. All antibodies were purchased from Cell Signaling Technologies (Danvers, MA, USA) except anti-caspase-3 (Abcam, Cambridge, UK) and anti-GAPDH (Merck Millipore, Burlington, MA, USA) antibodies. Bands were detected using the Clarity Western ECL Substrate (Bio-Rad Laboratories) chemiluminescence system with a Vü-C (Pop-Bio IMAGING, Cambridge, UK) imaging system, and were quantified using Image Studio Lite Version 5.2 (LI-COR Biosciences, Lincoln, NE, USA). 

### 4.8. Quantitative PCR 

Total RNA isolation from DLD1 cells was performed using TRI Reagent (Molecular Research Center, Cincinnati, OH, USA). The RNA concentration and purity were determined using NanoDrop One (Thermo Fisher Scientific). Synthesis of cDNA was performed using Maxima First Strand cDNA Synthesis Kit for RT-qPCR (Thermo Fisher Scientific) according to the manufacturer’s protocol. Quantitative PCR (qPCR) was performed using SsoAdvanced Universal SYBR Green Supermix (Bio-Rad Laboratories) in the CFX Connect Real-Time Detection System (Bio-Rad Laboratories). Data were analyzed using CFX Manager Software (Bio-Rad Laboratories) and LinRegPCR software [48]. All Ct values were normalized to geometrical means of rpl13a Ct values. Data are presented as relative changes, obtained using the ∆∆Ct method [49]. The list of primers is presented in Table 2.

### 4.9. In Vivo Experiment 

**Figure 9:**
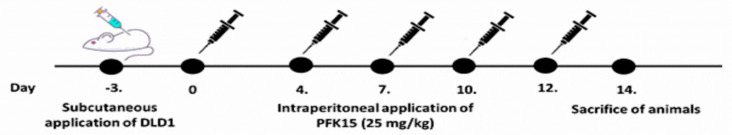
Experimental in vivo procedure for PFK15 administration. Nude mice bearing xenografts were intraperitoneally treated with PFK15 at 25 mg/kg a total of five times over 2 weeks. Animals were sacrificed on day 14.

The experiment was approved by the Institutional Ethics Committee of the Biomedical Research Center, Slovak Academy of Sciences, Bratislava and the State Veterinary and Food Administration of the Slovak Republic (Project Registration No. Ro 1289/18 - 221) in compliance with the Directive 2010/63/EU and the Regulation 377/2012 on the protection of animals used for scientific purposes in the approved animal facility (License No. SK UCH 02017). Male athymic nude mice (NU(NCr)-Foxn1nu) were purchased from Charles River (Velaz, Czech Republic). The mice were housed in sterile individually ventilated cages (room temperature 24 °C, 12:12 LD cycle, lights on at 7:00 h). Sterilized water and food were provided ad libitum. Tumor DLD1 cells were collected from the exponential growth phase and resuspended in PBS. Mice were injected subcutaneously with 0.1 mL of cell suspension (4 × 10^6^ cells) to the left and right site of the body (5 animals/untreated group; 6 animals/treated group). Monitoring of tumor growth was performed twice per week using a Vernier caliper. Tumor mass was determined according to the following formula: mass (mg) = (width, mm)^2^ × (length, mm)/2 [6]. After 3 days, mice were intraperitoneally treated with PFK15 at 25 mg/kg or a vehicle for 14 days (Figure 9). PFK15 administration was performed at two different times: morning group, 2 h after the onset of a light phase (ZT 02); evening group, injected 2 h before the onset of a dark phase (ZT 10). The mice were monitored daily, and their body weight was recorded before each PFK15 or vehicle injection. At the indicated time, mice were sacrificed by cervical dislocation. Tumors were harvested for histology and additional molecular analysis according to standard procedures. 

### 4.10. Tissue Specimens

The tissue specimens were fixed in 10% neutral buffered formalin and paraffin embedded for histological examination with hematoxylin and eosin staining. 

### 4.11. Immunohistochemistry 

Paraffin blocks of formalin-fixed tissues were cut into 4 µm thick serial slices. The Ki-67 antigen and PECAM-1 (CD31) were detected immunohistochemically. Monoclonal mouse anti-human Ki-67 antigen (clone MIB-1, isotype IgG1, kappa; Agilent Technologies, Inc., Dako, Glostrup, Denmark) was used at a dilution of 1:100, and monoclonal rabbit anti-mouse PECAM-1 (CD31) antibody (EPR17259; ab182981, Abcam) at a dilution of 1:2000 (incubation time: 20 min at room temperature using an automated immunostainer; Dako Autostainer Plus S 3400; Dako). Antigen enhancement (epitope retrieval) was performed by immersing the slides in citrate buffer (Dako EnVisionTM FLEX Target Retrieval Solution, low pH, 50×) and heating (in a steamer as well as using the automatic Dako PT Link Pre-Treatment Module for Autostainer; Dako). A conjugated reporter enzyme (peroxidase) and polymer (EnVision; Dako) [50] were used for detection with a chromogenic (diaminobenzidine) substrate (H_2_O_2_), then tissues were counterstained with hematoxylin. The prepared histological slides were semiquantitatively evaluated using an optical microscope (Leica DM2000; Leica Microsystems, Mannheim, Germany). The Ki-67 staining was given an immunoreactive score based on the intensity of nuclear staining and the number of cells stained according to a subjective grading system [51]. The staining intensity was classified into four categories, with the most strongly staining case as the upper limit: 0, negative; 1, weakly positive; 2, moderately positive; 3, strongly positive. The quantity of stained cells was scored according to the percentage of stained tumor nuclei: 0, 0%; 1, 1–10%; 2, 11–50%; 3, 51–80%; 4, >80%. The total immunoreactive score was expressed as a product of the scores obtained for staining intensity and quantity. A total score of 0 was considered as negative, 1–3 as weak, 4–6 as moderate, and 8–12 as strong immunoreactivity.

### 4.12. Tumor Parenchyma Cell Regressive Changes Morphometry

The prepared histological slides were evaluated by optical microscope (Leica DM2000, Leica Microsystems, Mannheim, Germany) semiquantitatively. The quantity (proportion, area) of cell regressive changes was scored as follows: 0%, 1–10%, 11–25%, 26–49%, ≥50% of tumor parenchyma cell regressive changed proportion/area in the field of view at low magnification of the microscope.

### 4.13. Statistical Analysis

The results are presented as mean ± SEM, and each value represents the average of at least three wells from at least three independent experiments. Normality was tested by Shapiro–Wilk test. Statistical differences among groups were determined by one-way ANOVA followed by Tukey’s post hoc test or by Kruskal–Wallis test. A *p*-value <0.05 was considered statistically significant. Statistical analyses were performed using STATISTICA 7.0 (StatSoft Inc., Tulsa, OK, USA) and GraphPad Prism 6 (GraphPad Software, Inc., San Diego, CA, USA). 

## 5. Conclusions

In conclusion, our study shows that PFK15 has a potent antiproliferative effect on cancer cells derived from colorectal carcinoma and on endothelial cells, as the viability of both cell types was reduced after PFK15 treatment. The effects of PFK15 are mediated via suppression of Akt protein kinase expression and its phosphorylation. Moreover, the treatment decreased the Bcl-2/Bax ratio, suggesting that the anticancer effects of PFK15 are associated with an increased level of apoptosis, which was proved by flow cytometry. Our findings demonstrate that glycolysis inhibition is a promising strategy for cancer treatment, as PFK15 effectively reduced tumor growth after morning but not evening treatment in our animal model. Moreover, our data suggest that chronotherapy can be an effective approach in cancer treatment, but circadian variation in target processes should be considered. 

## Figures and Tables

**Table 1:** Expression of Ki-67 in control (*n* = 5) and morning and evening PFK15-treated groups (*n* = 6). A total score of 0 was considered as negative, 1–3 as weak, 4–6 as moderate, and 8–12 as strong reaction.

Extent of Ki-67 IHC Reaction	Ki-67 Immunoreactivity, Number of Positive Reaction (%)			
	Negative	Weak	Moderate	Strong
Control group	0	0	0	100
Morning group	0	0	50	50
Evening group	0	0	17	83

**Table 2:** List of primers.

p21	Forward	5′-CGATGGAACTTCGACTTTGTCA-3′
	Reverse	5′-GCACAAGGGTACAAGACAGTG-3′
cyclin B1	Forward	5′-AACTTTCGCCTGAGCCTATTTT-3′
	Reverse	5′-TTGGTCTGACTGCTTGCTCT-3′
cyclin D1	Forward	5′-CAATGACCCCGCACGATTTC-3′
	Reverse	5′-CATGGAGGGCGGATTGGAA-3′
rpl13a	Forward	5′-GGACCGTGCGAGGTATGCT-3′
	Reverse	5′-ATGCCGTCAAACACCTTGAGA-3′

## Data Availability

Not applicable.
